# Levosimendan for Weaning From Veno-Arterial Extracorporeal Membrane Oxygenation in Cardiogenic Shock: An Umbrella Review

**DOI:** 10.7759/cureus.114745

**Published:** 2026-08-18

**Authors:** Abhishek Hanumanpratap Singh Kshatri, Minahil Saif, Semil Saleem, Muhammad Habib, Muhammad Zauraiz Malik, Azlaan Hussain, Samia Arif

**Affiliations:** 1 Industrial Health, ESIC Medical College and PGIMSR, Kalaburagi, IND; 2 Medicine, Maharajah's Institute of Medical Sciences, Vizianagaram, IND; 3 Emergency Medicine, Apollo Hospitals Health City, Visakhapatnam, IND; 4 Medicine, King Edward Medical University, Lahore, PAK; 5 Medicine and Surgery, King Edward Medical University, Lahore, PAK; 6 Medicine and Surgery, Mayo Hospital, Lahore, PAK

**Keywords:** amstar 2, cardiogenic shock, inotropic therapy, levosimendan, mechanical circulatory support, umbrella review, va-ecmo, va-ecmo weaning

## Abstract

Veno-arterial extracorporeal membrane oxygenation (VA-ECMO) is a key rescue therapy in cardiogenic shock (CS), but weaning failure remains common and is associated with worse outcomes. Levosimendan, a calcium-sensitizing inotrope with vasodilatory and cardioprotective properties, has been proposed to facilitate weaning, and several systematic reviews and meta-analyses with overlapping primary studies have examined this question, with varying conclusions regarding the strength and consistency of the evidence. This umbrella review was conducted to consolidate that evidence base.

Following PRISMA 2020 and PRIOR guidelines, PubMed, Embase, Scopus, and Cochrane Library were searched from inception to May 2026 for systematic reviews and meta-analyses evaluating levosimendan as an adjunct for VA-ECMO weaning in adult CS patients. Two reviewers independently screened studies, extracted data, and assessed methodological quality using AMSTAR 2; given substantial overlap in constituent primary studies, a narrative synthesis was performed rather than a de novo meta-analysis.

Seven reviews published between 2020 and 2025, pooling 5 to 18 primary studies and 557 to 2,274 patients, met inclusion criteria. All seven reviews found levosimendan significantly improved successful VA-ECMO weaning versus control (OR/RR range 1.20-2.89), with heterogeneity varying across reviews (I² 0-77%), and most also reported a significant reduction in mortality. Secondary outcomes were less consistent, with two reviews reporting numerically longer VA-ECMO support duration or ICU stay in levosimendan-treated patients.

AMSTAR 2 confidence ratings ranged from low (n=2) to moderate (n=4) to high (n=1), substantial primary-study overlap was identified across reviews, and GRADE certainty, where formally assessed, was rated very low. Levosimendan may be associated with improved VA-ECMO weaning success and a possible mortality benefit in CS, particularly in patients with combined cardiac insufficiency, but this concordance rests on overlapping, retrospective, non-randomized cohorts, making it hypothesis-generating rather than an evidence-based standard pending adequately powered randomized controlled trials.

## Introduction and background

Cardiogenic shock (CS) remains one of the most lethal conditions in contemporary cardiovascular medicine, with in-hospital mortality historically reported between 27% and 51% despite advances in revascularization and critical care [1]. Acute myocardial infarction accounts for the majority of CS cases, and population-level data from the CDC WONDER database confirm that CS-related mortality has continued to rise in heart failure populations over the past two decades, underscoring the persistent clinical burden of this syndrome [2]. Veno-arterial extracorporeal membrane oxygenation (VA-ECMO) has emerged as a cornerstone rescue therapy for patients with refractory CS that provides temporary biventricular pump and oxygenator support by draining venous blood, oxygenating it externally, and returning it to the arterial circulation, thereby offloading the heart while allowing time for myocardial recovery [3]. Its use has expanded substantially over the past decade as a salvage strategy for patients failing conventional pharmacologic management.

Despite its life-saving potential, successful liberation from VA-ECMO remains a major clinical challenge. Multicenter data from 23 tertiary centers across seven countries demonstrate that weaning fails in approximately 40% of patients placed on VA-ECMO for CS, and even among those successfully weaned, more than one-third die before hospital discharge [3]. Successful weaning refers to the ability to permanently discontinue ECMO support with adequate native cardiac output, typically assessed via a trial of reduced flow with echocardiographic and hemodynamic monitoring; this is distinct from survival to hospital discharge, since a substantial proportion of patients who wean successfully still die from ECMO-related complications or multiorgan failure before discharge. Predictors of weaning failure include older age, persistent cardiac arrest physiology, slow lactate clearance, reduced left ventricular ejection fraction, and prolonged ECMO duration beyond seven days [3,4]. These findings highlight an urgent need for pharmacologic strategies that can optimize myocardial recovery and facilitate timely device removal, since prolonged ECMO support is independently associated with bleeding, thromboembolic, and infectious complications that further worsen prognosis.

Levosimendan, a calcium-sensitizing inotrope with additional vasodilatory and cardioprotective properties, has been proposed as an adjunct to facilitate weaning from VA-ECMO by enhancing myocardial contractility without substantially increasing oxygen demand, owing to its long-acting active metabolite OR-1896 [5]. Over the past several years, this hypothesis has been tested in numerous primary observational studies, prompting several systematic reviews and meta-analyses that consistently, though with varying study pools and statistical approaches, report improved weaning success and, in several analyses, reduced mortality with levosimendan use [5-8]. However, substantial overlap exists in the primary studies these reviews incorporate, and their conclusions vary in strength, heterogeneity, and methodological rigor. Most recently, the LEVOECMO trial found that levosimendan did not improve 30-day successful VA-ECMO weaning or survival compared with placebo, and was associated with more ventricular arrhythmias [9]. An umbrella review, which systematically synthesizes existing systematic reviews and meta-analyses on a single question, is therefore warranted to consolidate this evidence, evaluate the consistency and quality of conclusions across reviews, and clarify the overall strength of evidence supporting levosimendan as a weaning strategy in VA-ECMO-supported CS patients.

## Review

Materials and methods

Study Selection

This umbrella review was conducted in accordance with the Preferred Reporting Items for Systematic Reviews and Meta-Analyses (PRISMA) 2020 guidelines and the PRIOR (Preferred Reporting Items for Overviews of Reviews) statement [10,11]. This umbrella review was not registered on PROSPERO or an equivalent registry. A comprehensive literature search was performed to identify systematic reviews and meta-analyses evaluating levosimendan as an adjunct for weaning from VA-ECMO in patients with CS. The search was designed to capture quantitative syntheses of observational studies and/or randomized controlled trials examining levosimendan use in this population. PubMed, Embase, Scopus, and Cochrane Library were systematically searched from inception until May 2026. The search strategy combined Medical Subject Headings (MeSH) and free-text keywords such as "levosimendan," "VA-ECMO," "veno-arterial extracorporeal membrane oxygenation," "cardiogenic shock," and "weaning". The search strategy used for PubMed was: “((Simendan[MeSH Terms]) OR (Levosimendan[Title/Abstract])) AND ((extracorporeal membrane oxygenation[MeSH Terms]) OR (extracorporeal membrane oxygenation[Title/Abstract]) OR (extracorporeal life support[Title/Abstract]) OR (ECMO[Title/Abstract]) OR (ECLS[Title/Abstract]))”. No language restrictions were applied. Reference lists of all included articles were manually screened to identify additional eligible reviews. Search results were imported into Rayyan for deduplication, followed by screening. Two reviewers (S.A. and S.S.) independently assessed titles and abstracts to determine potential eligibility, and full-text versions of potentially relevant articles were retrieved for detailed evaluation. Disagreements regarding inclusion were resolved through discussion and, if necessary, arbitration by a third reviewer (M.S.). The selection process was documented using a PRISMA flow diagram outlining the number of records identified, screened, excluded, and ultimately included in the final analysis.

Eligibility Criteria

Eligible studies included systematic reviews or meta-analyses that examined the use of levosimendan in adult patients supported on VA-ECMO for CS or refractory cardiac dysfunction, with or without concomitant mechanical ventilation (MV) weaning indications. To be considered, reviews must have included at least one observational study or randomized controlled trial involving human participants aged 18 years or older. Levosimendan exposure was defined broadly, including any dosing regimen, infusion rate, or timing relative to ECMO cannulation or weaning attempt. Reviews comparing levosimendan to standard therapy, traditional inotropes or vasopressors, milrinone, or no levosimendan administration were considered eligible. Only reviews that explicitly evaluated clinical outcomes such as successful weaning rate, mortality, ECMO duration, or length of ICU/hospital stay were included. Reviews that did not report VA-ECMO-specific outcomes separately from other mechanical circulatory support modalities, narrative reviews, scoping reviews, conference abstracts, and reviews lacking a systematic methodology or a clearly defined literature search were excluded. Articles focused solely on preclinical, animal, or in vitro studies were also excluded, as was gray literature.

Data Extraction

Data were independently extracted from each included review by two reviewers (M.S. and M.H.) using a pre-designed, standardized extraction form. Variables included first author, year of publication, number and design of included primary studies (observational vs. randomized), total sample size, population characteristics, comparator interventions, and reported primary and secondary outcomes. For reviews reporting pooled effect estimates, extracted data also included odds ratios, risk ratios, mean differences, 95% confidence intervals, p-values, I² values for heterogeneity, and details of subgroup, sensitivity, or dose-stratified analyses where performed. Information on protocol registration and risk-of-bias tools used by each review (e.g., Newcastle-Ottawa Scale, ROBINS-I, and Cochrane RoB) was also recorded [12-14]. Any discrepancies in data extraction were resolved through discussion or with input from a third reviewer.

Quality Assessment

The methodological quality of all included systematic reviews and meta-analyses was assessed using the AMSTAR 2 (A Measurement Tool to Assess Systematic Reviews) checklist [15]. This tool evaluates systematic reviews across 16 domains, including protocol registration, comprehensiveness of the literature search, duplicate study selection and data extraction, risk-of-bias assessment of primary studies, appropriateness of meta-analytic methods, accounting for risk of bias in result interpretation, and assessment of publication bias. Each review was independently rated by two reviewers (S.Y. and S.A.) as high, moderate, low, or critically low confidence based on the presence or absence of critical and non-critical flaws per AMSTAR 2 guidance. Disagreements in scoring were resolved through discussion and consensus.

Data Analysis

Given that this umbrella review synthesized previously published systematic reviews and meta-analyses with substantial overlap in their constituent primary studies, a de novo meta-analysis was not conducted. Instead, a narrative synthesis of findings across reviews was performed, focusing on the direction, magnitude, and consistency of treatment effects for successful VA-ECMO weaning and mortality, contextualized alongside heterogeneity estimates (I²), GRADE certainty ratings where reported, and AMSTAR 2 confidence ratings. Given the recurrence of several frequently cited primary cohorts across the seven included reviews, the degree of primary-study overlap was qualitatively assessed by cross-referencing reference lists; a formal corrected covered area (CCA) calculation was not performed by any of the included reviews and is noted as a limitation rather than computed independently in this umbrella synthesis.

Results

Study Selection Process

**Figure 1:**
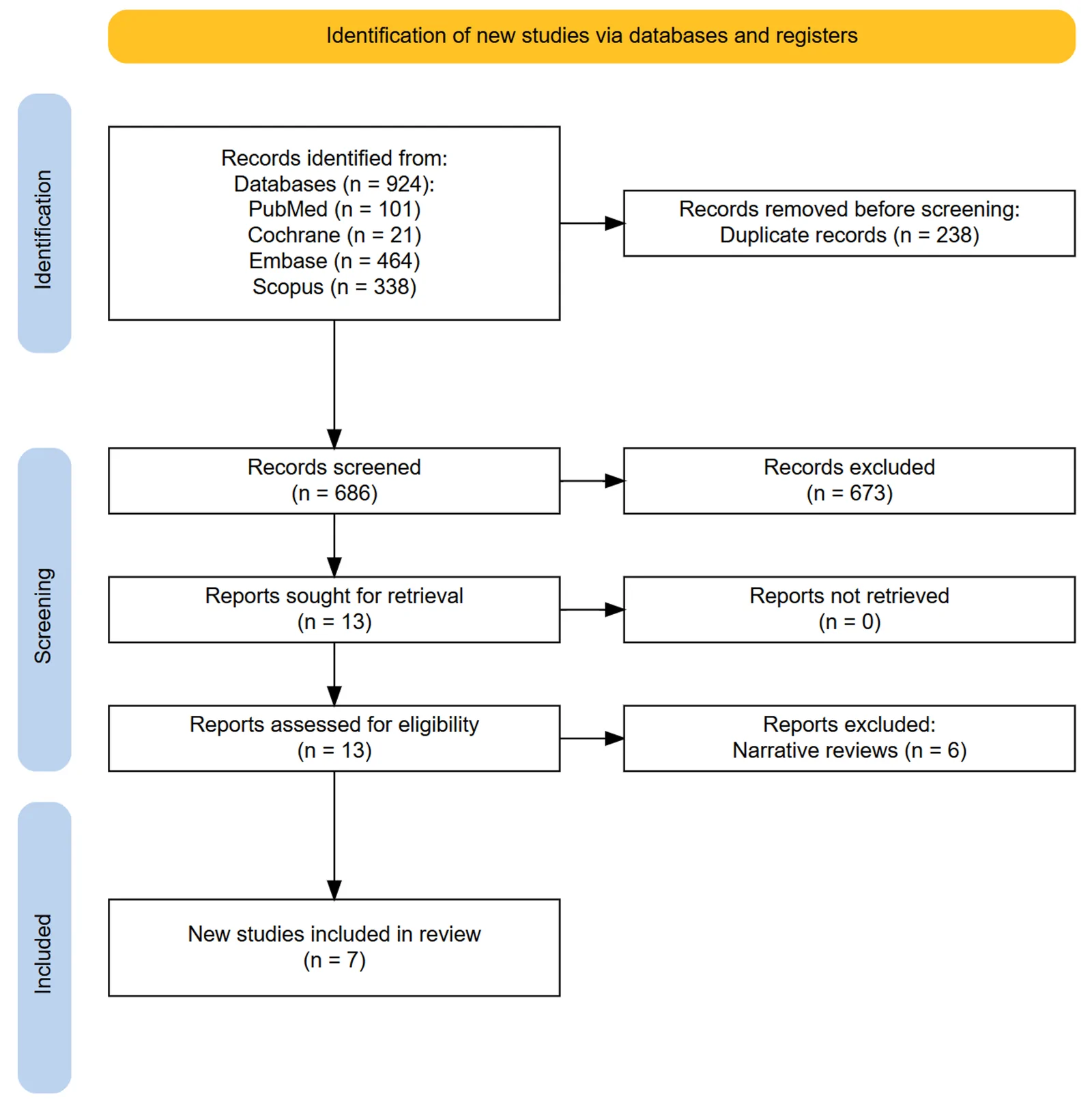
PRISMA diagram illustrating the study selection process. PRISMA, Preferred Reporting Items for Systematic Reviews and Meta-Analyses

A total of 924 records were initially retrieved through systematic searches across PubMed, Embase, Scopus, and Cochrane Library. After the removal of 238 duplicate entries, 686 unique records remained for screening. Following the initial screening phase, 13 articles were identified as potentially eligible and were retrieved for full-text evaluation. Upon thorough assessment against the predefined inclusion and exclusion criteria, seven systematic reviews and meta-analyses met all eligibility requirements and were included in the final umbrella review [5-8,16-18]. The remaining six full-text articles were excluded as they were narrative reviews. No additional studies were identified through manual screening of the reference lists of the included reviews or related publications. The entire selection process is summarized in the PRISMA flow diagram (Figure 1).

Study Characteristics

Seven systematic reviews and meta-analyses, published between 2020 and 2025, were included in this umbrella review [5-8,16-18]. Collectively, these reviews synthesized data from a combined pool of 5 to 18 primary studies each, encompassing a total patient population ranging from 557 to 2,274 across the individual reviews. The primary studies underpinning these reviews were predominantly observational in design, comprising retrospective and prospective cohort studies, before-after studies, registries, and case series conducted largely at single centers; only the review by Luo et al. incorporated a mix of observational studies and randomized controlled trials (14 observational studies and 4 RCTs), while the remaining four reviews relied exclusively on non-randomized, observational evidence [7].

The included populations across all seven reviews consisted of critically ill adult patients with CS or refractory cardiac dysfunction supported on VA-ECMO, with Luo et al. additionally including patients weaning from MV [7]. The intervention under investigation was consistently levosimendan, compared against heterogeneous control regimens across reviews, including standard therapy, traditional inotropes or vasopressors, milrinone, or no levosimendan administration (Table 1).

**Table 1:** Characteristics of included studies evaluating levosimendan for weaning from VA-ECMO in cardiogenic shock. VA-ECMO, veno-arterial extracorporeal membrane oxygenation; MV, mechanical ventilation; RCTs, randomized controlled trials

Author	Year	Studies included	Sample size	Included study designs	Population	Intervention	Comparison
Zhao et al. [18]	2025	16 studies	2,083	Retrospective cohort studies	Adults with CS supported on VA-ECMO	Levosimendan	Standard therapy or Placebo
Bertini et al. [17]	2023	10 studies	987	Observational studies	Adults (one pediatric cohort) with CS supported on VA-ECMO	Levosimendan	Standard therapy or Placebo
Liu et al. [6]	2024	15 studies	1,772	Observational studies	Critically ill adult patients receiving VA-ECMO	Levosimendan	Standard therapy or Placebo
Luo et al. [7]	2021	18 studies	2,274	14 observational + 4 RCTs	Adults weaning from MV or VA-ECMO	Levosimendan	Standard therapy or Placebo
Kaddoura et al. [5]	2021	7 studies	630	Observational studies	Critically ill adult patients on VA-ECMO	Levosimendan	Standard therapy or Placebo
Burgos et al. [16]	2020	5 studies	557	Retrospective cohort studies	Adult patients with cardiogenic shock treated with VA-ECMO	Levosimendan	Standard therapy or Placebo
Yang et al. [8]	2021	9 studies	1,058	Observational studies	Adult patients receiving VA-ECMO for refractory cardiogenic shock or cardiac arrest	Levosimendan	Standard therapy or Placebo

The primary outcome assessed uniformly across all seven reviews was the successful weaning rate from VA-ECMO (or MV/ECMO, where applicable). Secondary outcomes varied but commonly included all-cause or in-hospital mortality, ECMO duration, length of ICU or hospital stay, vasopressor or vasoactive drug use, adverse events, and hemodynamic or echocardiographic parameters; Burgos et al. assessed all-cause mortality as its sole secondary outcome, while Yang et al. focused on in-hospital mortality alone [8,16]. The main findings of the included studies are summarized in the following table (Table 2).

**Table 2:** Summary of primary and secondary outcomes, main findings, and conclusions reported by included systematic reviews and meta-analyses on levosimendan use during weaning from VA-ECMO in cardiogenic shock. MV, mechanical ventilation; ECMO, extracorporeal membrane oxygenation; VA-ECMO, veno-arterial extracorporeal membrane oxygenation; WMD, weighted mean difference; OR, odds ratio; RR, risk ratio; CI, confidence interval; I², heterogeneity index; ICU, intensive care unit; LOS, length of stay; LVEF, left ventricular ejection fraction; IABP, intra-aortic balloon pump; NNT, number needed to treat; GRADE, Grading of Recommendations Assessment, Development and Evaluation; RCTs, randomized controlled trials

Author	Primary outcomes	Secondary outcomes	Main findings	Conclusions
Zhao et al. [18]	Successful weaning rate from MV/ECMO	30-day mortality; in-hospital mortality; duration of VA-ECMO support; ICU length of stay	Levosimendan significantly improved VA-ECMO weaning success (OR=2.44, 95% CI 1.72-3.48, p<0.00001, I^2^=57%). It reduced 30-day mortality (OR=0.48, 95% CI 0.29-0.81, p=0.006) and in-hospital mortality (OR=0.47, 95 CI 0.26-0.88, p=0.02). However, it was associated with prolonged VA-ECMO support (WMD=2.86 days, 95% CI 1.73-4.00) and longer ICU stay (WMD=5.69 days, 95% CI 2.19-9.20). Benefit on weaning was consistent across subgroups (infusion rate, etiology, sample size).	Levosimendan improves VA-ECMO weaning success and reduces short-term mortality, but is linked to longer ECMO support and ICU stay. Given the retrospective, single-center design of included studies and residual risk of selection bias, further high-quality RCTs are required to confirm benefits and define its optimal clinical role.
Bertini et al. [17]	Successful weaning rate from MV/ECMO	Mortality; ICU/hospital length of stay; bleeding risk; ECMO duration; renal replacement therapy; peak creatinine; adverse drug events	Levosimendan significantly associated with successful weaning (362/448 vs. 328/539; OR 2.37, 95% CI 1.71-3.28, p=0.001, I²=0%) and reduced mortality (144/433 vs. 258/507; OR 0.53, 95% CI 0.36-0.78, p=0.01, I²=39%). Secondary outcomes (LOS, bleeding, ECMO duration, RRT, creatinine) showed no significant differences; no adverse drug events reported.	Levosimendan administration may help reduce the risk of failure in weaning from VA-ECMO while conferring a survival benefit; however, evidence is based solely on observational studies, and further RCTs are needed to confirm efficacy.
Luo et al. [7]	Successful weaning rate from MV/ECMO	Mortality, duration of MV/ECMO, length of ICU stay, adverse events	Levosimendan improved MV/ECMO weaning (OR=2.32, 95% CI 1.60-3.36, p<0.00001, I²=68%); benefit was significant in low LVEF/MV patients, ECMO after cardiac surgery, and ECMO with cardiogenic shock, but not in MV weaning-difficulty patients; ECMO subgroup showed reduced mortality (OR=0.66) but no MV mortality benefit; no significant differences in MV/ECMO duration, ICU LOS, or adverse events	Levosimendan significantly increases successful weaning from cardiopulmonary support, particularly in patients with combined cardiac insufficiency; large, well-designed RCTs are needed to better define the patient subgroups most likely to benefit
Burgos et al. [16]	Successful weaning rate from MV/ECMO	All-cause mortality	Levosimendan significantly increased successful VA ECMO weaning vs. controls (RR=1.42, 95% CI 1.12-1.80, p=0.004, I²=71%); pooled weaning prevalence was 61.4% (95% CI 39.8-82.9%); levosimendan was also associated with decreased risk of all-cause mortality (RR=0.62, 95% CI 0.44-0.88, p=0.007, I²=36%); pooled mortality prevalence was 36% (95% CI 29.6-48.8%)	Levosimendan may facilitate VA ECMO weaning and reduce all-cause mortality in adult cardiogenic shock patients; few studies are available, and prospective randomized multicenter trials are warranted to confirm these benefits
Kaddoura et al. [5]	Successful weaning rate from MV/ECMO	All-cause mortality, length of ICU/hospital stay, vasopressor use, hemodynamic/echocardiographic parameters	Levosimendan was significantly associated with successful VA-ECMO weaning vs. control (OR=2.89, 95% CI 1.53-5.46, p=0.001, I²=49%); sensitivity analysis excluding 2 high-risk studies showed comparable results (OR=3.64, 95% CI 1.59-8.33); for mortality, 6 studies (n=620) showed significantly lower risk with levosimendan (OR=0.46, 95% CI 0.30-0.71, p=0.0004, I²=20%, NNT=5); GRADE certainty was very low for both outcomes; IABP use during weaning was significantly lower with levosimendan	Levosimendan therapy was significantly associated with successful weaning and survival benefit in patients with cardiogenic or postcardiotomy shock needing VA-ECMO; evidence remains limited and of very low certainty, with no randomized trial data available, so these findings should be considered hypothesis-generating pending confirmatory RCTs
Liu et al. [6]	Successful weaning rate from MV/ECMO	All-cause mortality, ECMO duration, length of ICU/hospital stay, vasopressor use	Levosimendan significantly increased weaning success vs. control (OR=2.78, 95%CI 1.80-4.30, p<0.00001, I²=65%), with less heterogeneity in post-cardiac surgery patients (OR=2.06, 95%CI 1.35-3.12, I²=17%); benefit was significant only at 0.2 mcg/kg/min dosing (OR=2.45, 95%CI 1.11-5.40); 1-month mortality was significantly reduced with levosimendan (OR=0.47, 95%CI 0.28-0.79, p=0.004, I²=73%); ECMO duration was longer in the levosimendan group (non-significant, p=0.05, I²=77%); vasoactive drug use (norepinephrine, dobutamine, adrenaline) was higher in some studies in the levosimendan group	In patients receiving VA-ECMO, levosimendan treatment considerably raised the weaning success rate and helped lower mortality without increasing it; since most evidence comes from retrospective studies, more randomized multicenter trials are required to verify the conclusion
Yang et al. [8]	Successful weaning rate from MV/ECMO	In-hospital mortality	Levosimendan significantly increased weaning incidence from VA-ECMO (79.3% vs. 63.4%; RR=1.20, 95% CI 1.07-1.34, p=0.002, I²=49%); Levosimendan significantly reduced in-hospital mortality vs. control (46.3% vs. 50.7%; RR=0.80, 95% CI 0.67-0.95, p=0.013, I²=31%); one-way sensitivity analyses confirmed stability of both findings, and funnel plots showed low probability of publication bias for both outcomes	Levosimendan use significantly increased the incidence of weaning in patients undergoing VA-ECMO and reduced in-hospital mortality; however, given the wide range of clinical and methodological variability across included observational studies, the true effect of levosimendan could not be definitively established, and RCTs are still required

Quality Assessment

The AMSTAR 2 quality assessment revealed variable methodological rigor across the seven included systematic reviews and meta-analyses, with confidence ratings ranging from low to high (Table 3). One review was rated high confidence, reflecting a comprehensive multi-database and grey-literature search, use of the ROBINS-I tool for risk-of-bias assessment, GRADE-based evaluation of evidence certainty, and formal testing for publication bias [5]. Four reviews were rated moderate confidence, having satisfied all critical domains without outright failures but showing partial weaknesses such as narrower search strategies, incomplete reporting of excluded studies, and, in two cases, absence of a formal GRADE or risk-of-bias-impact assessment [6,7,17,18]. The remaining two reviews were rated low confidence due to a single critical-domain failure each, namely absence of a registered protocol in one case and lack of formal publication-bias assessment in the other, compounded by additional non-critical limitations [8,16]. No review was excluded from the umbrella review on the basis of quality assessment, given the relatively limited and evolving body of evidence on levosimendan for VA-ECMO weaning; instead, these methodological limitations are considered when interpreting the consistency and strength of the pooled findings reported across reviews.

**Table 3:** AMSTAR 2 quality assessment of included studies. PICO, population, intervention, comparator, outcome; PROSPERO, International Prospective Register of Systematic Reviews; RoB, risk of bias; NOS, Newcastle-Ottawa Scale; ROBINS-I, Risk Of Bias In Non-randomized Studies of Interventions; GRADE, Grading of Recommendations Assessment, Development and Evaluation

AMSTAR 2 item	Zhao et al. [18]	Bertini et al. [17]	Burgos et al. [16]	Kaddoura et al. [5]	Liu et al. [6]	Luo et al. [7]	Yang et al. [8]
1. PICO components stated	Yes	Yes	Yes	Yes	Yes	Yes	Yes
2. Pre-registered protocol (PROSPERO/other)	Yes	Yes	Yes	Yes	Yes	Yes	No
3. Explanation of study design choice	No	No	No	No	No	No	No
4. Comprehensive literature search	Partial Yes	Yes	Partial Yes	Yes	Partial Yes	Yes	Partial Yes
5. Study selection in duplicate	Yes	Yes	Yes	Yes	Yes	Yes	Yes
6. Data extraction in duplicate	Yes	Yes	Yes	Yes	Yes	Yes	Yes
7. List of excluded studies with reasons	Partial Yes	Partial Yes	Partial Yes	Yes	Partial Yes	Partial Yes	Partial Yes
8. Adequate description of included studies	Yes	Yes	Yes	Yes	Yes	Yes	Yes
9. Satisfactory RoB assessment technique	Yes (NOS)	Yes (ROBINS-I)	Yes (NOS)	Yes (ROBINS-I)	Yes (NOS)	Yes (Cochrane RoB + NOS)	Yes (NOS)
10. Funding sources of included studies reported	No	No	Yes	Yes	Yes	No	Yes
11. Appropriate meta-analytical methods	Yes	Yes	Yes	Yes	Yes	Yes	Yes
12. Assessment of RoB impact on results	No	Yes (GRADE)	No	Yes (GRADE)	No	No	No
13. RoB accounted for in interpretation/discussion	Partial Yes	Yes	Partial Yes	Yes	Partial Yes	Partial Yes	Partial Yes
14. Satisfactory explanation of heterogeneity	Yes	Yes	Yes	Yes	Yes	Yes	Yes
15. Investigation of publication bias	Yes (funnel + Begg's/Egger's + trim-and-fill)	Yes (funnel + Egger's test)	No (too few studies)	Yes (funnel plots)	Yes (funnel plots)	Yes (funnel plots)	Yes (funnel plots)
16. Conflicts of interest disclosed	Yes	Yes	Yes	Yes	Yes	Yes	Yes
Overall confidence rating	Moderate	Moderate	Low	High	Moderate	Moderate	Low

Discussion

This umbrella review synthesized findings from seven systematic reviews and meta-analyses examining levosimendan use for weaning from VA-ECMO in patients with CS, collectively encompassing 5 to 18 primary studies and sample sizes ranging from 557 to 2,274 patients [5-8,16-18]. Despite differences in inclusion criteria, search dates, and analytical approaches, all seven reviews converged on a consistent direction of effect: levosimendan was associated with significantly improved successful weaning from VA-ECMO compared with control therapy, with odds or risk ratios ranging from 1.20 to 2.89, and most reviews also reported a significant reduction in mortality [5,6,8,16-18].

The seven included reviews varied considerably in scope and primary studies pooled. Burgos et al. restricted their analysis to five non-randomized retrospective studies (557 patients), reporting a pooled weaning success risk ratio of 1.42 (95% CI 1.12-1.80) and a mortality risk ratio of 0.62 (95% CI 0.44-0.88) [16]. Kaddoura et al., published shortly afterward, expanded the evidence base to seven observational studies (630 patients) and found a stronger weaning association (OR 2.89, 95% CI 1.53-5.46) alongside a mortality benefit (OR 0.46, 95% CI 0.30-0.71), though the GRADE certainty for both outcomes was rated very low due to confounding and inconsistency [5]. Bertini et al., pooling 10 observational studies (987 patients, including one pediatric cohort), similarly reported a significant weaning benefit (OR 2.37, 95% CI 1.71-3.28, I² 0%) and a mortality benefit (OR 0.53, 95% CI 0.36-0.78), with no significant differences across secondary outcomes including length of stay, bleeding risk, ECMO duration, or renal replacement therapy [17]. Liu et al. further broadened inclusion to 15 studies and 1,772 patients, again confirming a weaning benefit (OR 2.78, 95% CI 1.80-4.30) and mortality reduction (OR 0.47, 95% CI 0.28-0.79), while introducing dose-stratified subgroup analysis suggesting that an infusion rate of 0.2 mcg/kg/min, rather than 0.1 mcg/kg/min, was associated with significant weaning benefit [6]. Zhao et al., the most recent and second-largest review (16 studies, 2,083 patients), likewise confirmed a significant weaning benefit (OR 2.44, 95% CI 1.72-3.48) and reductions in both 30-day mortality (OR 0.48, 95% CI 0.29-0.81) and in-hospital mortality (OR 0.47, 95% CI 0.26-0.88), though levosimendan was associated with prolonged VA-ECMO support (WMD 2.86 days, 95% CI 1.73-4.00) and longer ICU stay (WMD 5.69 days, 95% CI 2.19-9.20) [18]. Luo et al. took a broader cardiopulmonary support perspective, pooling both ECMO and MV weaning studies (18 studies, 2,274 patients). This study also found an overall weaning benefit (OR 2.32, 95% CI 1.60-3.36), though subgroup analysis revealed this benefit was confined to ECMO patients and to MV patients with reduced left ventricular ejection fraction, with no benefit observed in patients with isolated MV weaning difficulty unrelated to cardiac dysfunction [7]. Yang et al., focusing exclusively on VA-ECMO (nine studies, 1,058 patients), reported a more conservative but still significant effect on both weaning (RR 1.20, 95% CI 1.07-1.34) and in-hospital mortality (RR 0.80, 95% CI 0.67-0.95), with sensitivity analyses confirming stability of these findings [8].

A notable feature across all seven reviews is the substantial overlap in primary studies. Several frequently cited cohorts appear repeatedly across reviews, meaning that the apparent concordance in pooled effect estimates partly reflects shared rather than independent evidence [19-22]. This overlap is a defining methodological feature of umbrella reviews of overlapping meta-analyses and warrants caution when interpreting the cumulative weight of evidence, since the CCA for primary study overlap was not formally calculated in any of the seven included reviews.

The proposed mechanistic rationale for the effect of levosimendan was consistent across reviews. As a calcium sensitizer, levosimendan enhances myocardial contractility by increasing the calcium sensitivity of troponin C, without raising intracellular calcium or myocardial oxygen demand. Although its opening of ATP-sensitive potassium channels in vascular smooth muscle produces systemic, pulmonary, and coronary vasodilation, reducing both right and left ventricular afterload, these complementary inotropic and vasodilatory actions, combined with the prolonged activity of its metabolite OR-1896, have been proposed as the pharmacological basis for its use in facilitating gradual weaning from mechanical circulatory support [23]. Liu et al. and Kaddoura et al. further noted dose-dependent associations, with 0.2 mcg/kg/min appearing more consistently beneficial than lower infusion rates, although the limited number of studies precluded definitive dose-response conclusions [5,6].

Several limitations recur across the seven reviews and merit emphasis in this umbrella synthesis. First, nearly all primary studies were single-center, retrospective, and non-randomized, exposing the pooled estimates to substantial risk of confounding by indication; sicker patients may have preferentially received levosimendan, or conversely, only patients deemed to have recovery potential may have been selected for treatment [5-8,16-18]. Kaddoura et al. explicitly rated their evidence as "very low" certainty using GRADE, citing critical risk of bias due to confounding across all included studies [5]. The retrospective design of nearly all constituent studies means treatment allocation was never randomized, so measured and unmeasured prognostic factors, such as baseline ventricular function, lactate trajectory, or timing of ECMO initiation, could differ systematically between levosimendan-treated and control patients in ways that standard covariate adjustment cannot fully correct. Selection bias compounds this concern: clinicians may have preferentially initiated levosimendan in patients judged to have residual myocardial reserve and a realistic chance of weaning, while excluding those perceived as too unstable to tolerate it, which would artificially inflate the apparent benefit of the drug rather than reflect a true causal effect. Conversely, if levosimendan was reserved as a last-resort agent in the most refractory patients, its benefit could be underestimated, creating a bidirectional uncertainty that cannot be resolved without randomization. Because every included review shares this same underlying threat, the concordant direction of effect across reviews should not be mistaken for independent corroboration of a causal relationship; rather, it may represent a consistent, shared bias replicated across overlapping observational cohorts. These considerations directly temper the overall conclusions of this umbrella review: the consistency of association across reviews increases confidence that levosimendan is correlated with improved weaning outcomes in current practice, but it does not establish that levosimendan causes this improvement, and the true treatment effect could be smaller than, or in a different direction from, that reported by the pooled observational estimates. Second, heterogeneity in the definition of "successful weaning" persisted across primary studies, with some defining failure as death during ECMO support or within 24 hours of removal and others using different timeframes or criteria altogether, complicating direct comparison [5,8,16]. Third, dosing regimens, timing of levosimendan initiation relative to ECMO cannulation or weaning attempt, and comparator groups (ranging from no intervention to traditional inotropes to milrinone) varied substantially, introducing clinical heterogeneity that likely contributed to the statistical heterogeneity (I² values ranging from 0% to 77%) observed across outcomes [5-8,16-18]. Fourth, secondary outcomes such as ECMO duration and length of ICU or hospital stay were less consistently favorable, with Liu et al., Luo et al. MV subgroup analyses, and Zhao et al. all noting numerically longer support durations or ICU stays in levosimendan-treated patients, possibly reflecting either a true prolongation effect of the drug's vasodilatory properties or confounding by indication in sicker patients selected for treatment [6,7,18].

Collectively, this body of overlapping evidence suggests a plausible and mechanistically coherent benefit of levosimendan in facilitating VA-ECMO weaning and reducing short-term mortality, particularly in patients with combined cardiac insufficiency [7]. However, the consistent absence of randomized controlled trial data across all seven reviews, the very low GRADE certainty ratings where formally assessed, and the substantial primary study overlap mean these findings should be regarded as hypothesis-generating rather than practice-defining [5].

Clinical implications and future directions

Given the consistent direction of effect across all seven included systematic reviews, levosimendan may be reasonably considered as an adjunctive pharmacological strategy when attempting VA-ECMO weaning in patients with CS, particularly those with documented left ventricular systolic dysfunction or combined cardiac insufficiency, where the strongest and most consistent subgroup benefit was observed. Clinicians should, however, weigh this potential benefit against the very low certainty of evidence, the absence of any randomized controlled trial data, and the substantial overlap of primary studies across reviews. Until confirmatory trial data emerge, levosimendan use for this indication should be regarded as an individualized, case-by-case decision rather than a standardized protocol, ideally restricted to centers with experience in its hemodynamic monitoring and dosing. Subsequent studies should standardize the definition of successful weaning, report dosing and timing of administration relative to cannulation, and stratify outcomes by ECMO indication and ejection fraction to clarify which patient subgroups derive genuine benefit. Future systematic reviews should also formally calculate and report CCA to quantify primary study overlap, improving transparency in this rapidly accumulating but methodologically overlapping evidence base.

## Conclusions

This umbrella review of seven overlapping systematic reviews and meta-analyses indicates that levosimendan is consistently associated with improved successful weaning from VA-ECMO and reduced short-term mortality in patients with CS, with the strongest signal observed in those with combined cardiac insufficiency. However, this concordance rests on a narrow, repeatedly recycled base of retrospective, single-center, non-randomized cohorts, with very low GRADE certainty where formally assessed. Levosimendan therefore represents a biologically plausible, hypothesis-generating adjunct to current weaning strategies rather than an evidence-based standard of care. Definitive guidance awaits adequately powered randomized controlled trials, which alone can establish causality and clarify which patient subgroups derive genuine benefit.
